# Catalyst-Free Crosslinking Modification of Nata-de-Coco-Based Bacterial Cellulose Nanofibres Using Citric Acid for Biomedical Applications

**DOI:** 10.3390/polym13172966

**Published:** 2021-08-31

**Authors:** Rabiu Salihu, Mohamed Nainar Mohamed Ansari, Saiful Izwan Abd Razak, Nurliyana Ahmad Zawawi, Shafinaz Shahir, Mohd Helmi Sani, Muhammad Hanif Ramlee, Mohammed Ahmad Wsoo, Abdul Halim Mohd Yusof, Nadirul Hasraf Mat Nayan, Ahmad Mohammed Gumel

**Affiliations:** 1 Department of Biosciences, Faculty of Science, Universiti Teknologi Malaysia, Skudai 81300, Johor, Malaysia; salihu.r@fud.edu.ng (R.S.); nurliyana@utm.my (N.A.Z.); shafinazshahir@utm.my (S.S.); helmisani@utm.my (M.H.S.); mohammed1981@uor.edu.krd (M.A.W.); 2 Bioinspired Device and Tissue Engineering Research Group, Faculty of Engineering, School of Biomedical Engineering and Health Sciences, Universiti Teknologi Malaysia, Skudai 81310, Johor, Malaysia; 3 Department of Microbiology and Biotechnology, Federal University Dutse, PMB 7156 Ibrahim Aliyu Bypass, Dutse 720101, Jigawa State, Nigeria; am.gumel@fud.edu.ng; 4 Institute of Power Engineering, Universiti Tenaga Nasional, Kajang 43000, Selangor, Malaysia; 5 Medical Devices and Technology Centre (MEDiTEC), Institute of Human-Centered Engineering (iHmEn), Universiti Teknologi Malaysia, Skudai 81300, Johor, Malaysia; m.hanif@utm.my; 6 Department of Chemistry, College of Science, University of Raparin, Rania 46012, Kurdistan Region, Iraq; 7 Faculty of Engineering, School of Chemical and Energy Engineering, Universiti Teknologi Malaysia, Skudai 81300, Johor, Malaysia; halimy@cheme.utm.my; 8 Faculty of Engineering Technology, Universiti Tun Hussein Onn Malaysia, Batu Pahat, Bahru 86400, Johor, Malaysia; nadirul@uthm.edu.my

**Keywords:** bacterial cellulose, citric acid, catalyst-free, crosslinking, nata-de-coco, biomedicine

## Abstract

Bacterial cellulose (BC) has gained attention among researchers in materials science and bio-medicine due to its fascinating properties. However, BC’s fibre collapse phenomenon (i.e., its inability to reabsorb water after dehydration) is one of the drawbacks that limit its potential. To overcome this, a catalyst-free thermal crosslinking reaction was employed to modify BC using citric acid (CA) without compromising its biocompatibility. FTIR, XRD, SEM/EDX, TGA, and tensile analysis were carried out to evaluate the properties of the modified BC (MBC). The results confirm the fibre crosslinking phenomenon and the improvement of some properties that could be advantageous for various applications. The modified nanofibre displayed an improved crystallinity and thermal stability with increased water absorption/swelling and tensile modulus. The MBC reported here can be used for wound dressings and tissue scaffolding.

## 1. Introduction

The Philippines-originated jelly dessert (nata de coco) is the cheapest form of bacterial cellulose (BC) produced through the fermentation of coconut water [1]. It is a pure form of (BC) with unique physicochemical, morphological, and mechanical properties [1,2]. Owing to this, nata de coco-based BC can serve as a good reference point for applications such as biomedicine, where high material purity is a primary demand. Also, its large-scale production in many Asian countries like Malaysia, Indonesia, and Thailand [3] is due to the ease of the processes.

BC is a microfibrillar biomaterial first reported by Andrian Brown in 1886. Different species of bacteria such as *Acetobacter xylinum*, *Rhizobium*, *Achromobacter*, and *Sarcina* have been reported to produce BC through fermentation [4,5,6]. BC is generally pure, bio-compatible, and non-toxic, and it can be modified into a broad range of different forms and compositions. This means it has a remarkable range of applications in many fields of science and medicine [7,8,9,10]. However, its inability to reabsorb much water after being dehydrated and its inadequate functionality limit its attractiveness. Hence, there is a need to modify it further [11].

In situ and ex situ modifications were the principal approaches for BC modification. Ex situ modification is usually done after BC production and involves either physical or chemical processes [12,13,14]. One of the methods of interest is the crosslinking reaction (a process that induces a strong link between polymer chains through covalent bonding), owing to its simplicity and effectiveness [15].

Citric acid (CA) is one of the organic acids classified as “generally regarded as safe” (GRAS) by the US food and drug administration (FDA) that has long been used as a modifier on many polymeric biomaterials via crosslinking, including BC [16,17,18,19,20]. Modifying polymeric biomaterials with CA typically yields what is known as citrate-based biomaterials (CBBs) [21,22]. Owing to their excellent biological, chemical, and material properties, (e.g., antimicrobial, antioxidant, and fluorescence properties), CBBs have been used increasingly frequently in biomedicine [23,24,25]. Crosslinking a biopolymer with CA requires elevated temperatures of 120–190 °C [26]. This method has the advantage of allowing the material’s mechanical, chemical, and degradation properties (among other properties) to be fine-tuned [27,28].

The CA crosslinking of biopolymers involving different catalysts has been reported by many scientists, but the undesirable effects posed by the catalysts [29,30] have limited their application in biomedicine. Therefore, we hypothesised that the use of the catalyst for BC crosslinking modification is unnecessary.

In this study, a catalyst-free thermal crosslinking approach was employed for the first time to modify nata de coco-based BC fibres using a readily available and inexpensive multifunctional monomer (i.e., CA). An evaluation of the physicochemical, morphological, and mechanical properties of the resulting biopolymer showed that it retained some of its important properties (e.g., thermal stability) while exhibiting a better crystallinity index, water absorption rate, and tensile modulus than unmodified BC. Our method seems to be the cheapest and easiest approach that yields promising improvements to BC’s properties. Moreover, the findings show that the use of a catalyst for BC modification might be unnecessary.

## 2. Materials and Methods

### 2.1. Chemicals and Materials

Bacterial cellulose (BC) sheets were purchased from a local nata de coco company (Happy Alliance). CA monohydrate powder (C_6_H_8_O_7_·H_2_O) and sodium hydroxide (NaOH) were all purchased from Merck (Sigma-Aldrich, St. Louis, MI, USA).

### 2.2. Purification and Modification of Bacterial Cellulose (BC)

BC hydrogels were modified with CA by thermal crosslinking as in [31] with slight modifications to exclude the catalyst. Briefly, the wet BC sample was cut into 100 mm × 50 mm pieces and purified in 1% (*w*/*v*) NaOH at 90 °C for 60 min before being washed with distilled water until the pH reached 7–8 at 37 °C. BC sheets were immersed in different molar (M) concentrations (0.0375, 0.075, 0.15, 0.3, and 0.6) of a CA solution in ion-exchanged distilled water (diH_2_O) and allowed to stand for 24 h at 45 °C. They were then cured in an oven at 140 °C for 2 h. Another BC sample with the same dimensions was treated under the same conditions using only diH_2_O—this sample served as the control sample. All samples were then removed and rinsed with diH_2_O until the pH reached 5–6. After this, the samples were tagged as BC (pristine), MBC0.03 (0.0375 M), MBC0.07 (0.075 M), MBC0.15 (0.15 M), MBC0.30 (0.3 M), or MBC0.60 (0.6 M), and freeze-dried for characterisation.

### 2.3. Characterisation

For comparison, the BC films were characterised based on their physicochemical, morphological, and mechanical properties through scanning electron microscopy (SEM), energy-dispersive X-ray (EDX), Fourier transform infrared (FTIR), X-ray diffraction (XRD), water contact angle (WCA), swelling rate (SR), thermogravimetric analysis (TGA), and tensile modulus.

#### 2.3.1. Scanning Electron Microscopy (SEM)

The surface morphology of the fibres before and after modification was examined by SEM analysis (Model: Hitachi TM3000, Tokyo, Japan) equipped with an EDX system. Micrographs of platinum sputter-coated samples were taken at an accelerating voltage of 15 kV for different magnifications.

#### 2.3.2. Fourier Transformed Infrared (FTIR)

The BC, MBC samples were analysed using (Model: PerkinElmer-Frontier™, L1280044, Waltham, MA, USA) spectrophotometer equipped with an attenuated total reflection (ATR-FTIR) system as in [32]. The spectra were obtained from scans between a wavelength range of 4000 to 650 cm^−1^ and 4 cm^−1^ resolutions.

#### 2.3.3. X-ray Diffraction (XRD)

The XRD analysis was performed using an X-ray diffractometer (Model: Rigaku SmartLab, Austin, TX, USA.) with a CuKα radiation wavelength (λ = 0.154 nm) operated at 40 kV and 30 mA. Scans were undertaken between angle 2θ values of 10° to 60°at a speed of 3°/min. The crystallinity index (CrI) was calculated based on values obtained from the peaks analysis on origin software using Equation (1), while the crystallite size (CS), and the crystal allomorphs of cellulose I were calculated from the XRD data using Equations (2) and (3), respectively [33].

(1) $$\mathrm{CrI}\left(\%\right)=\mathrm{Area}\;\mathrm{of}\;\mathrm{crystalline}\;\mathrm{peaks}/\mathrm{Area}\;\mathrm{of}\;\mathrm{all}\;\mathrm{peaks}\;\left(\mathrm{crystalline}+\mathrm{amorphous}\right)\times 100\%$$

CS = Kλ/FWHMcosθ(2)

K is the Scherrer’s constant (0.9), λ is the X-ray wavelength (1.54 Å), FWHM is the width of the diffraction peak at maximum height, and θ is the Bragg’s angle.

*Z* = 1693d_1_ − 902d_2_ − 594 (3)

The term Z denotes discriminant function developed by [34], d_1_ is the d-spacing of 1-10 peak, and d_2_ is the d-spacing of 110 peaks, where Z > 0 or Z < 0 indicates I_α_ or I_β_ rich type of cellulose, respectively [35].

#### 2.3.4. Water Contact Angle (WAC)

An Optima machine (Model: 1020046094) equipped with a camera was used to capture water droplet images and measure the WCA. Briefly, 20 × 20 mm sheets were cut after freeze-drying before this process was performed. A uniform droplet of 2.0 µL of deionised water (diH_2_O) was dispensed on five (5) different points on each sample, and the average angle was then recorded [36].

#### 2.3.5. Swelling Rate (SR)

Freeze-dried samples were cut into 30 × 30 mm pieces. Their dried weight (W_1_) values were then recorded before they were immersed in either distilled water or simulated body fluid (SBF) at ambient temperature. Samples were then removed and weighed at specific intervals after having been blotted with filter paper to remove excess water until an equilibrium weight (W_2_) was reached. The swelling rate was calculated using Equation (4) below [37,38].

SR = (W_2_ − W_1_)/W_1_ × 100% (4)

where W_1_ is the dried weight and W_2_ is the final wetted weight.

#### 2.3.6. Thermal Gravimetric Analysis (TGA)

The thermal stability of all samples was evaluated using a thermal analyser (Shimadzu DTG-60H, Kyoto, Japan). For all the samples, a freeze-dried film weighing 18 ± 3 mg was heated in a platinum pan to 30 °C to 900 °C at a heating rate of 10°C/min under a nitrogen atmosphere of 100 mL/min flow rate. The weight loss upon heating was normalised as percentage weight loss (%) and plotted against the corresponding temperature (°C) [39].

#### 2.3.7. Tensile Properties

The tensile properties of the BC and MBC samples were evaluated using a tensile testing machine (Zwick/Roell Z020, Zwick, Ulm, Germany) according to ASTM-D882 standards. Briefly, freeze-dried samples that were kept in a desiccator were cut into a rectangular shape (70 × 20 mm) with different thicknesses and a gauge” length of 50 mm. Samples were strained at maximum load of 2.0 kN and a crosshead speed of 2 mm/min. The stress was determined as loading force in Newton (N) against the cross-sectional area (width × thickness) of the sample and the strain. The strain was calculated as ΔL/L_0_ where ΔL is the exerted extension from starting point and L_0_ is the initial length [40,41] The modulus was then determined from the linear region of the 0.2% offset of the stress/strain curve. All measurements were performed for at least five samples under ambient conditions.

## 3. Results and Discussion

### 3.1. Scanning Electron Microscopy (SEM)

**Figure 1:**
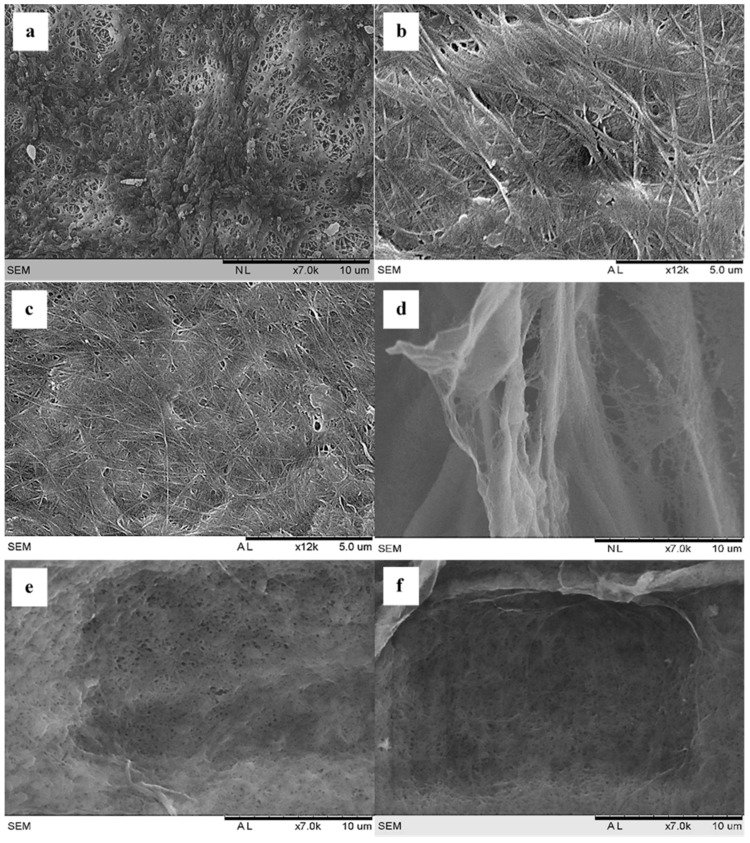
Scanning electron microscope (SEM) images for (**a**) bacterial cellulose (BC), (**b**) MBC0.03, (**c**) MBC0.07, (**d**) MBC0.15, (**e**) MBC0.30, and (**f**) MBC0.60.

In the SEM micrographs shown in Figure 1, cellulose fibres can be observed with different surface morphologies among the samples. The unmodified sample (BC) appears to have a compacted fibre network with uniformly interconnected pores similar to what [42] reported. The modified samples, on the other hand, displayed different fibre networks depending on the CA concentration. At lower concentrations (MBC0.03, MBC0.07, and MBC0.15), porous fibre networks can be observed, which could allow for more water absorption. Higher concentrations (MBC0.30 and MBC0.70), in contrast, showed compacted fibres, similar to the untreated sample. This could be due to the high crosslinking density between the fibres, thus affecting the porosity and preventing the passage of water molecules and leading to a low swelling rate (as explained in Section 3.5 and Figure 6). This could also be why [29] obtained a different result since they used percentage concentrations, whereas the present study used molar concentrations of CA.

### 3.2. Fourier Transformed Infrared (FTIR)

**Figure 2:**
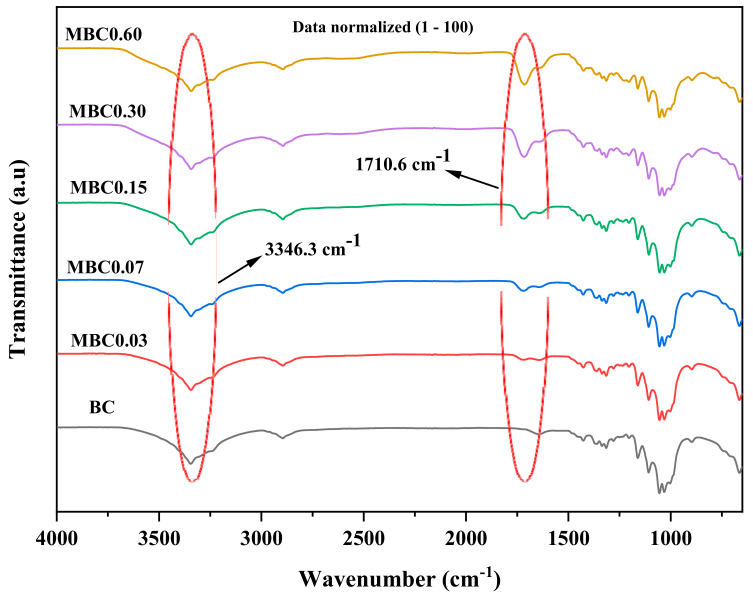
Fourier transform infrared (FTIR) spectrum of the unmodified and modified samples at different citric acid (CA) concentrations.

**Figure 3:**
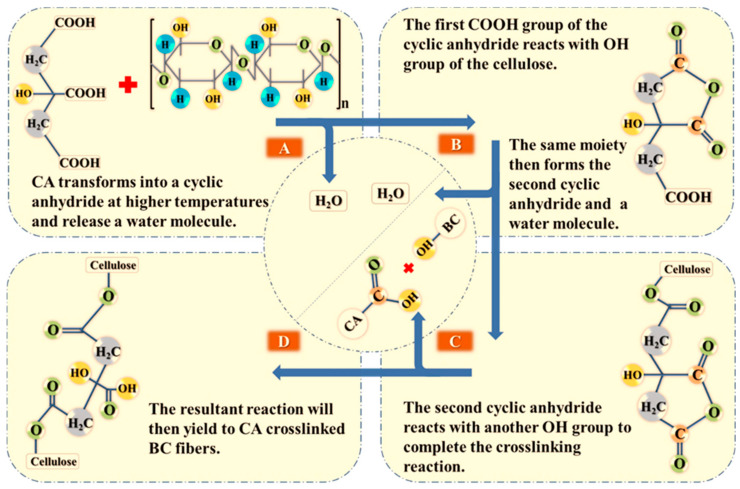
Schematic diagram of the proposed CA crosslinking mechanism on BC.

The FTIR spectra of the pristine and modified samples are shown in Figure 2. The signature peaks attributed to the dominant functional group of BC’s (OH-stretching) vibration were located at 3346 cm^−1^. Peaks at 2865 cm^−1^ and 1420 cm^−1^ were due to C–H stretching, and the peak at 1450 cm^−1^ was due to CH2 absorption. Peaks at 1719 cm^−1^ were related to carbonyl/carboxyl (C=O) stretching [43] and appear only on the crosslinked samples, thus confirming the presence of CA within the modified BC samples [31,44,45]. Peaks between 1055 cm^−1^ and 1020 cm^−1^ were due to C–O–C interactions. The reduced intensity of the OH peaks on the crosslinked samples can also result from the chemical interaction between cellulose and CA [46]. Overall, the increase in the intensity of the peaks associated to C=O-stretching (1710.6 cm^−1^) with increasing concentration of CA on treated samples indicate that crosslinking modification on the BC was successful. The proposed mechanism of CA crosslinking on BC was presented in a schematic diagram in Figure 3.

### 3.3. X-ray Diffraction (XRD)

**Figure 4:**
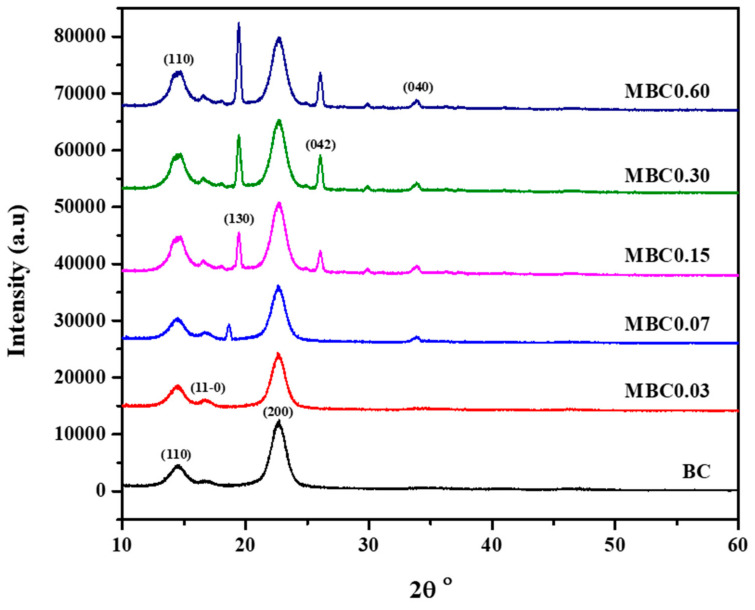
X-ray diffraction (XRD) spectra of the unmodified and modified BC.

The XRD patterns shown in Figure 4 represent the spectra obtained for the pure and modified samples. All samples showed peaks typical of cellulose I allomorph at lattice planes of 110, 1–10, and 200, corresponding to 2θ values of 14.6°, 16.6°, and 22.6°, respectively, as previously reported [33,35,47,48]. Distinctive peaks with different intensities were obtained at diffraction planes of 130, 042, and 040, corresponding to 2θ values of 19.4°, 26.1° and 34.3°. These peaks appeared only on the modified samples and, thus, were attributed to the CA crosslinking of the BC [49].

The peaks associated with BC’s crystallinity appeared with similar intensities for all samples, indicating that CA modification has a positive effect on the crystalline structure and morphology of BC [50,51]. Even though [16] reported a decrease in crystalline peaks on sodium carboxymethylcellulose (NaCMC) crosslinked with CA, such findings are likely due to one of the cellulose derivatives used. De Lima et al. observed decreased crystallinity and ascribed it to the increased viscosity of NaCMC or its interaction with cellulose nanofibres during crystallisation [52].

The calculated crystallinity indexes and crystallite size values are presented in Table 1. These values essentially show that the CA crosslinking has improved the crystallinity and crystallite size of the MBC. Furthermore, the cellulose I allomorph (calculated using the Z-discriminant function) showed that all the samples have the same cellulose I_α_ rich (triclinic) form, which is typical of bacterial cellulose [53,54,55]. All other calculated values aligned with previously reported data [31] and indicate that crosslinking modification with CA has improved the BC’s crystallinity.

### 3.4. Water Contact Angle (WCA)

**Figure 5:**
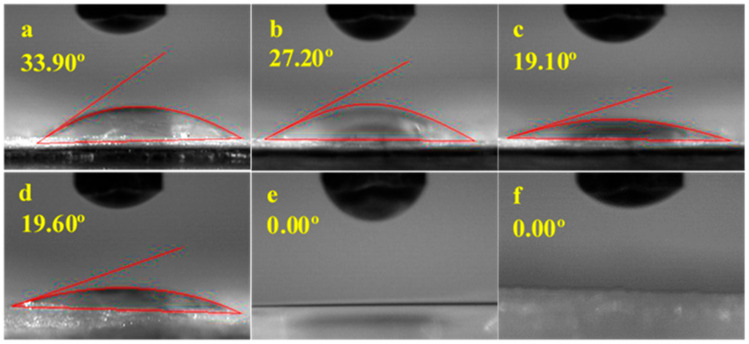
Mean water contact angles obtained for (**a**) BC, (**b**) MBC0.03, (**c**) MBC0.07, (**d**) MBC0.15, (**e**) MBC0.30, and (**f**) MBC0.60.

The wetting behaviour of a material’s surface is closely related to the molecular terminal groups present and contact angle studies give information about the wettability properties of materials [56]. In theory, a surface is considered hydrophilic or super hydrophilic when its WCA is below 90°or 10°, respectively [36]. Figure 5 depicts the mean contact angles measured for the pure and modified BC samples. All samples, including the pure BC sample, had WCAs between 0° and 33.90°, signifying that all samples were either hydrophilic or super hydrophilic.

However, it is noteworthy that the modified samples have shown decreasing WCAs of as low as 0° (MBC0.30 and MBC0.60), in which case water droplets are no longer capturable (they disperse as soon as they are dropped). BC’s hydrophilicity could be attributed to the additional carboxyl groups [43,57] that can form hydrogen bonds with water molecules [58]. Even though a native BC is inherently hydrophilic, the WCA tends to decrease with increasing the CA concentration. Essentially, the CA modification performed on BC in the present study has improved its surface chemistry to attract more water.

### 3.5. Swelling Rate (SR)

**Figure 6:**
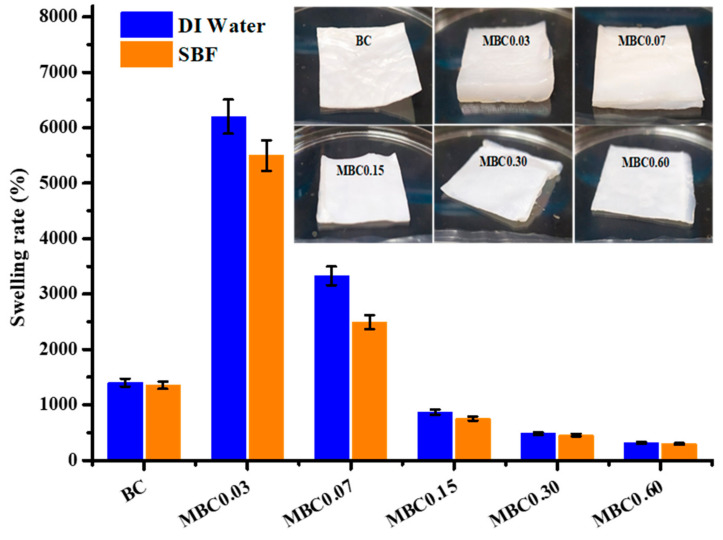
Swelling rates and sample images of the unmodified and modified BC after soaking in deionised (DI) water and simulated body fluid (SBF).

Generally, polymeric materials’ water absorption and swelling behaviour occur through capillary action and diffusion and the electrostatic repulsion between the ions on the polymer chains that force them to expand and swell [57]. The swelling rates (SRs) of pure and modified BC samples are presented in Figure 6.

Modified samples typically presented increased SRs at lower CA concentrations and decreased SRs (even dropping below that of pure BC) at higher CA concentrations. This decrease in SR could be due to the numerous crosslinker points formed within BC’s fibre networks, thus reducing the amount of space for water to enter [58]. It is evident from the samples with the lowest absorption rates (i.e., MBC0.30 and MBC0.60) that there is a packed fibre geometry on the SEM micrographs (in Figure 1), which could result from the high concentration of CA. The sample images in Figure 6 serve as additional evidence of the SR trend between samples at different CA concentrations.

Water absorption/swelling is especially advantageous for BC’s medical applications, such as its use as wound dressings [29]. The use of SBF to determine SR is mainly based on ascertaining whether the differences in the ionic concentrations of deionised water and SBF can affect the SR results. Interestingly, all samples in the present study showed similar SRs, both for SBF and deionised water. The SR results reported here corroborate a previous report that BC’s water holding capacity is 60 to 700 times its dry weight [59].

### 3.6. Thermal Gravimetric Analysis (TGA)

**Figure 7:**
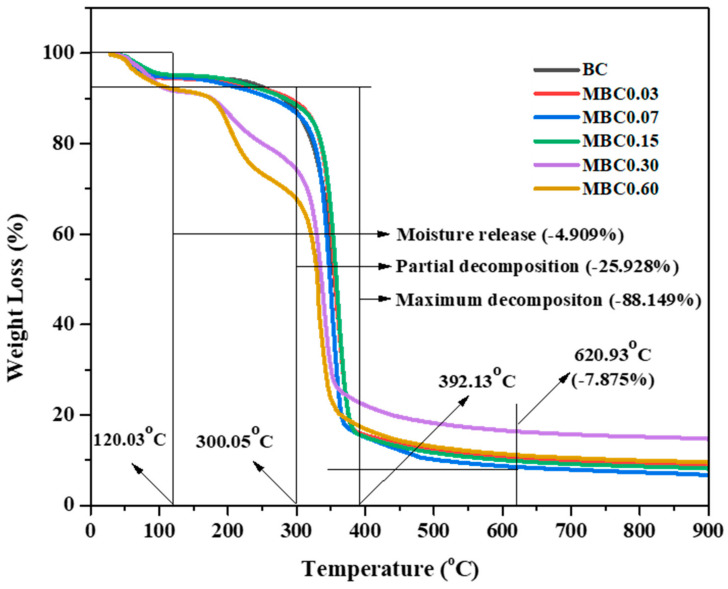
Thermal gravimetric analysis (TGA) graphs of the unmodified and modified BC samples.

An important property of BC is its thermal stability, especially when it is used in biomedicine applications, where higher temperatures are applied for sterilisation processes. Figure 7 shows the thermal behaviour of the pristine and modified BC evaluated in the present study.

The initial weight loss observed for all samples at temperatures of 45–120 °C was due to absorbed moisture evaporation. Except for the samples with the highest CA concentrations (MBC0.30 and MBC0.60), which displayed a partial decomposition of 120–300 °C, all modified samples were not different from the pristine BC. They all showed a maximum weight loss at 300–392 °C due to dehydration, decomposition, and the dissociation of glycosidic links [60,61,62]. The partial, total, and residual mass losses observed at maximum temperatures of 300 °C, 392 °C, and 620.93 °C were 25.928%, 88.149%, and 7.875%, respectively. The partial decomposition observed may also be due to the highest concentration of CA, which attracts more moisture than the lower concentrations. Our result implies that CA modification has little effect on the thermal properties of the BC [63].

### 3.7. Tensile Testing

**Figure 8:**
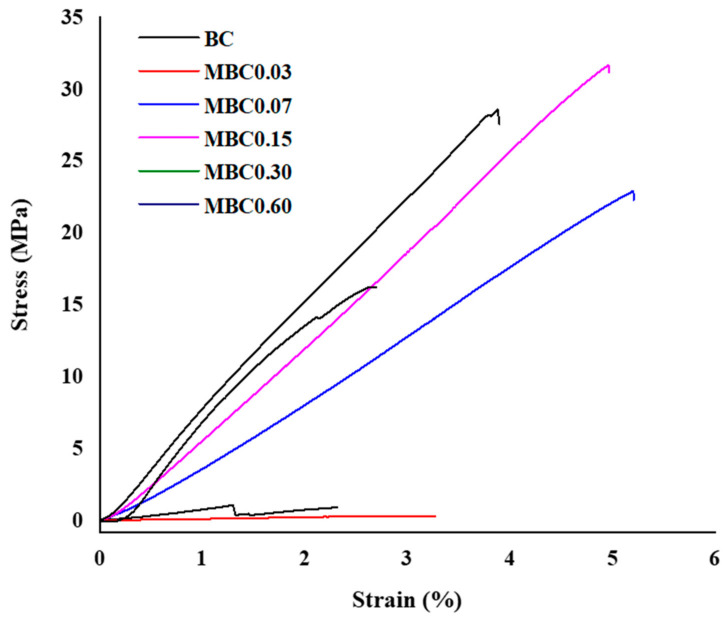
Mechanical properties of the modified and unmodified samples.

Table 2 presents the tensile test results and Figure 8 represents the stress/strain curves of all the samples. All modified samples exhibited better mechanical strength than the unmodified sample, except for the sample with the lowest CA concentration (MBC0.03), which exhibited a very low tensile strength value.

The decrease in the mechanical strength displayed could result from the smaller crosslinking degrees within the fibre networks due to the low amount of crosslinker. This supposition is supported by the SEM micrographs Figure 1. It can be observed that, despite having a lower modulus value, the elongation at the break is within the same range as observed in other modified samples. This implies that the elasticity of the fibres is close to that of the fibres in other modified samples after the maximum yield limit is reached.

Like the modulus, the tensile strength follows the trend of increasing as the CA concentration increases, except for the lowest concentration. However, the trend of elongation at the breaking point showed a different pattern, as it decreases as the CA concentration increases. Therefore, it can be hypothesised that applying a high-concentration CA treatment to BC may reduce the stretching ability of BC fibres.

## 4. Conclusions

Several attempts to enhance BCs’ properties through crosslinking modification involve the use of catalysts. However, some of these catalysts might alter the chemical composition and compromise the resulting polymer’s biocompatibility, thus limiting its application, especially in biomedicine. Here, we reported for the first time a catalyst-free modification of BC with CA using a simple immersion hydrothermal crosslinking method.

The improvements in the chemical, morphological, thermal, and mechanical properties presented in this report are an indication that the modification has resulted in a potential citrate-based biopolymer that can be used as wound dressings or tissue scaffold material. Although the cytotoxicity studies of the CA-modified BC is not within the scope of the current report, it is part of the authors’ future investigation. The approach used here seems to be the cheapest and easiest modification method that yields some promising results. Thus, the findings show that the proposed method is effective and that catalysts could be excluded from future BC modification techniques, especially those intended for biomedical applications.

## Figures and Tables

**Table 1:** Calculated crystallinity indexes and crystallite sizes of the modified and unmodified samples.

Sample	Crystallinity Index (%)	Crystallite Size (Å)	Allomorph
BC	80	51	Iα rich (triclinic)
MBC0.03	80	56	Iα rich (triclinic)
MBC0.60	81	56	Iα rich (triclinic)
MBC0.60	84	56	Iα rich (triclinic)
MBC0.60	91	56	Iα rich (triclinic)
MBC0.60	93	56	Iα rich (triclinic)

**Table 2:** Mechanical properties of the unmodified and modified samples as mean ± standard deviation.

Samples	Thickness (mm)	E_t_ (MPa)	_σ__M_ (MPa)	ε_B_ (%)
BC	0.99 ± 0.07	56.68 ± 7.81	1.25 ± 0.16	1.94 ± 0.06
MBC0.03	2.16 ± 0.19	17.97 ± 1.48	0.62 ± 0.16	3.97 ± 0.60
MBC0.07	0.16 ± 0.01	473.59 ± 62.02	20.60 ± 3.58	4.87 ± 0.25
MBC0.15	0.12 ± 0.01	778.42 ± 132.47	28.43 ± 3.15	4.11 ± 0.59
MBC0.30	0.08 ± 0.01	945.73 ± 199.62	26.65 ± 10.13	3.27 ± 0.71
MBC0.60	0.18 ± 0.09	1024 ± 44.66	16.37 ± 0.63	3.23 ± 0.36

## Data Availability

All data generated or analysed during the study are included in this article and are available from the corresponding author on reasonable request.
